# An ex vivo perfused ventilated murine lung model suggests lack of acute pulmonary toxicity of the potential novel anticancer agent (−)-englerin A

**DOI:** 10.1007/s00204-022-03235-z

**Published:** 2022-02-14

**Authors:** Christian Schremmer, Dirk Steinritz, Thomas Gudermann, David J. Beech, Alexander Dietrich

**Affiliations:** 1 Walther Straub Institute of Pharmacology and Toxicology, Member of the German Center for Lung Research (DZL), Medical Faculty, LMU-Munich, Nussbaum Str. 26, 80336 Munich, Germany; 2 Bundeswehr Institute of Pharmacology and Toxicology, Neuherbergstraße 11, 80937 Munich, Germany; 3 School of Medicine, University of Leeds, LIGHT Building, Clarendon Way, Leeds, LS2 9JT England, UK

**Keywords:** Anti-cancer drug, Lung edema, Classical transient receptor potential 4 and 5 (TRPC4, TRPC5), Tidal volume, Wet-to-dry weight ratio, FITC–dextran permeation assay

## Abstract

(−)-Englerin A (EA), a potential novel anti-cancer drug, is a potent selective activator of classical transient receptor potential 4 and 5 (TRPC4, TRPC5) channels. As TRPC4 channels are expressed and functional in the lung endothelium, possible side effects such as lung edema formation may arise during its administration. Well-established in vivo rodent models for toxicological testing, however, rapidly degrade this compound to its inactive derivative, englerin B. Therefore, we chose an ex vivo isolated perfused and ventilated murine lung (IPVML) model to detect edema formation due to toxicants, which also reduces the number of incriminating animal experiments required. To evaluate the sensitivity of the IPVML model, short-time (10 min) drops of the pH from 7.4 down to 4.0 were applied, which resulted in linear changes of tidal volumes, wet-to-dry weight ratios and incorporation of FITC-coupled dextran particles from the perfusate. As expected, biological activity of EA was preserved after perfusion in the IPVML model. Concentrations of 50–100 nM EA continuously perfused through the IPVML model did not change tidal volumes and lung weights significantly. Wet-to-dry weight ratios were increased after perfusion of 100 nM EA but permeation of FITC-coupled dextran particles from the perfusate to the lung tissues was not significantly different. Therefore, EA shows little or no significant acute pulmonary toxicity after application of doses expected to activate target ion channels and the IPVML is a sensitive powerful ex vivo model for evaluating acute lung toxicity in accordance with the 3R rules for animal experimentation.

## Introduction

(−)-Englerin A (EA) is a guaiane sesquiterpene originally isolated from the bark of the plant *phyllanthus engleri* from southern Africa (Wu et al. 2017). This natural product effectively inhibits growth of several kidney and breast cancer cell lines with an IC_50_ of 35–50 nM, while their non-tumorigenic counterparts were only affected at higher concentrations (> 10 µM) (Sourbier et al. 2013). Further analysis of cell lines revealed that EA is a potent activator of TRPC4 and TRPC5 channels and TRPC4 expression correlates with EA sensitivity of cancer cell lines (Akbulut et al. 2015; Carson et al. 2015). Both channels are members of the seven classical transient receptor potential family, which form homo- and heterotetrameric unselective cation channel. Death of synovial sarcoma (SW982) cells in vitro after application of EA (10 nM) occurs by Na^+^ influx through TRPC1/4 heteromeric channels and is further increased by inhibition of Na^+^/K^+^-ATPase removing excess intracellular Na^+^ ions (Ludlow et al. 2017; Muraki et al. 2017). Application of EA (2 mg/kg body weight) resulted in a significant reduction in locomotor activity for about 1 h as a potential side effect (Cheung et al. 2018). TRPC4- and TRPC5-deficient mice were partially and TRPC4/5 double knock-out mice were fully protected from this adverse EA effect (Cheung et al. 2018). In contrast to TRPC1/5, TRPC1/4 channels were also detected in murine lung endothelium (Sundivakkam et al. 2012) and are responsible for thrombin-induced lung edema, which is reduced in TRPC4-deficient lungs (Tiruppathi et al. 2002). Analysis of pulmonary toxicity of EA, however, turned out to be complicated for two reasons. First, intravenous (IV) injection of EA doses of > 1 mg/kg body weight were lethal in rats and second EA was rapidly degraded with an estimated half time of ≈ 15 min into the inactive compound englerin B in mice and rats by serum esterases, but not in human serum (Carson et al. 2015). The authors observed labored breathing in mice after subcutaneous injection of EA (5 mg/kg body weight) (Carson et al. 2015). It was, however, unclear, if this side effect was due to EA, which never exceeded levels of 12 nM in blood, or its metabolite englerin B, which was rapidly detected after application in rodents but not in humans. Therefore, we employed a new ex vivo model for evaluating acute EA toxicity using the isolated, perfused and ventilated murine lung (IPVML) offering numerous advantages compared to an in vivo system. We hypothesized that due to the absence of esterase in the perfusate EA is not degraded and defined doses can be applied. Lung parameters are analyzed in real time during the experiment without high stress levels in a living mouse. Moreover, wet to dry weight ratios and invasion of perfused fluorescein isothiocyanate (FITC)–dextran particle in the lung tissue can be quantified after the experiment. The applicability of the model was evaluated after applying short-time graduated pH changes from pH 7.4 to pH 4.0, which resulted in significant linear changes of values for wet-to-dry ratios and tidal volumes. Most interestingly, EA showed no significant changes in lung parameters and edema formation upon application of 50 or 100 nM EA [fivefold or tenfold of the EC_50_ values obtained in vitro experiments with SW982 cells (Muraki et al. 2017)] in comparison to control mice receiving electrolyte solution only.

## Materials and methods

### Chemicals

Electrolyte solution was prepared by the Apotheke Klinikum der Universität München: 7.19 g sodium chloride, 0.33 g potassium chloride, 0.27 g magnesium hexahydrate, 0.36 g calcium chloride dihydrate, 0.15 g potassium dihydrogen orthophosphate, 2.67 g glucose monohydrate, 51.28 g hydroxyethyl starch 200000/05 ad 1000 ml with aqua ad injectabilia, use 0.1848 mg/ml sodium hydrogen carbonate to adjust pH to 7.4. (−)-Englerin A (EA) was purchased from Roth (6492.1, Karlsruhe, Germany), Fluorescein isothiocyanate was provided from Sigma (90718, average Mw 70 kD, Taufkirchen, Germany) and GeneJuice^®^ Transfection Reagent was ordered from Sigma (70967, Taufkirchen, Germany). EA stock solution was prepared at 100 mM in the Novartis approved standard solution for all in-life pre-clinical evaluations containing 5% ethanol, 10% polyethylene glycol 300 (Sigma Aldrich), 5% cremophor EL (Merck Chemicals Ltd), and 80% PBS as described before (Carson et al. 2015; Cheung et al. 2018). The final concentration of this solution was 0.0001% in the electrolyte buffer.

### Isolated perfused and ventilated murine lung (IPVML)

IPVML were prepared from  C57BL/6 mice as described previously (Weissmann et al. 2006, 2012). Mice were anesthetized and anticoagulated by i.p. injection of ketamine (100 mg/kg body weight (bw), Covetrus, Hamburg, Germany), xylazine (7 mg/kg bw, Covetrus, Hamburg, Germany) and with heparin (500 U/kg bw, Meditech Vertriebs GmbH, Parchim, Germany). Lungs were transferred from the body into an artificial glass thorax, ventilated with room air (positive pressure ventilation, 90 breath/min) and perfused with the blood-free electrolyte solution heated to 37 °C. Upon reaching a final flow rate of 2 ml/min ventilation was switched to negative pressure (90 breath/min, 50% inspiration time) and a deep inspiration was initiated every 5 min*.* After 15 min of perfusion, an external reservoir (20 ml) containing electrolyte solution and indicated compounds (pH drop or EA) was connected and after an additional 4 min the perfusion circuit, which then contained only perfusion solution of the external reservoir, was closed. The pH was continuously measured in the external reservoir and adjusted, if necessary, in the first 10 min by a pH meter (WTW inoLab^®^ pH 7110, Weilheim, Germany). All data were transmitted to a computer and constantly monitored with the Pulmodyn software (Hugo Sachs Elektronik, March-Hugstetten, Germany). During perfusion with FITC–dextran particles, the solution was protected from light to avoid bleaching.

### Wet-to-dry weight ratios

For wet-to-dry weight ratios lung weight was quantified directly after the experiment (wet weight) and after drying at 50 °C for 48 h (dry weight).

### Ca^2+^ imaging

HEK293T cells were grown on Ø 25 mm coverslips for 24 h and transfected with a TRPC4β1 cDNA in a pIRES2 plasmid containing an eGFP cDNA under the control of the internal ribosome entry site (IRES) using GeneJuice^®^ solution as described by the manufacturer for 24 h. Cells were washed and loaded with FURA-2-AM (2 µM, F0888, Sigma, Taufkirchen, Germany) in 0,1% BSA in HEPES buffered HBSS at 37 °C for 30 min. Coverslips were washed with HEPES buffered HBSS and placed under a microscope in a recording chamber with a 500 µl volume. An increase in intracellular Ca^2+^ was recorded using a Leica DFC9000 GT camera coupled to an inverted microscope (DMi8, Leica, Wetzlar, Germany) with an 40x/0.85 oil immersions objective at 340 and 380 nm for quantification of [Ca^2+^]_i_ as described (Bendiks et al. 2020).

### Lung histology

Following perfusion with FITC–dextran particles mouse lungs were incubated in 4% (w/v) paraformaldehyde in PBS and processed for embedding in O.C.T™ Compound (Tissue-Tek^®^, Sakura Finetek, Umkirch, Germany) as described before (Weber et al. 2020). Cryo-embedded lungs were cut in 10 µm sections using a cryotome (CM1900, Leica, Wetzlar, Germany) and mounted on glass slides in Dako Omnis Fluorescence mounting medium (GM30411-2, Agilent Technologies, Santa Clara, USA). Lung sections were analyzed using both a confocal microscope (LSM 880, Carl Zeiss Jena, Germany) and a fluorescence scanner (Typhoon Trio, GE Healthcare, Solingen, Germany).

### Data analysis

Data analysis was performed using R and data were plotted using GraphPad Prism 9. Used statistical tests and p values are indicated in the figure legends.

## Results

### Experimental setup for detection of acute lung toxicity by ex vivo isolated perfused ventilated murine lungs (IPVML)

**Fig. 1:**
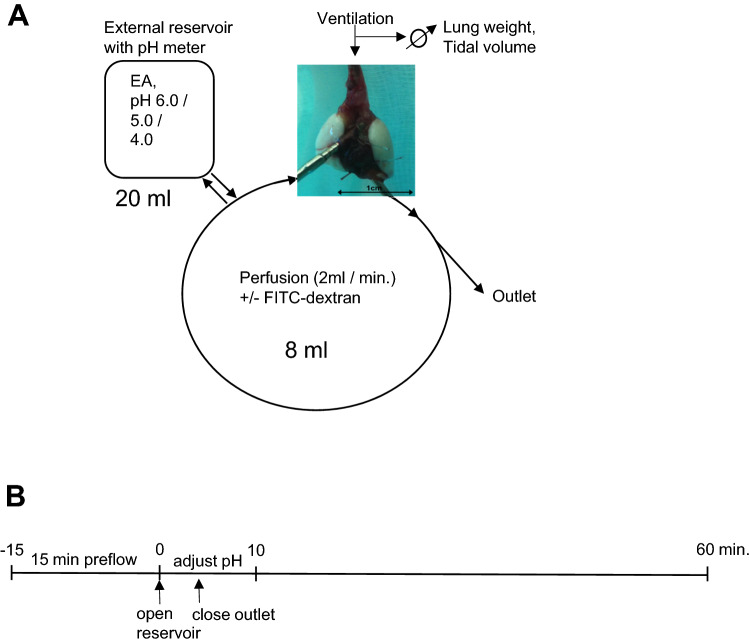
Experimental setup of the isolated perfused ventilated murine lung (IPVML) model (**A**). A freshly isolated lung heart preparation was perfused through the pulmonary artery and ventilated through the trachea. Twenty ml of solutions with Englerin A (EA, 50, 100 nM) or equilibrated to pH 6.0, pH 5.0 and pH 4.0 were relased from a reservoir in the perfusion solution. Lung weight and tidal volumes were continously quantified by in built weighing scales or the software, respectively. Time line of the experiment (**B**). After 15 min perfusion EA in solution or solutions of decreased pH were released from the reservoir. The pH was continously measured in the reservoir and adjusted for 10 min, if necessary. The outlet was closed after 4 min and the perfusion solutions circulated for another 50 min in a closed circuit

The experimental setup and the timeline for the detection of acute lung toxicity in the IPVML by edema formation are depicted in Fig. 1A, B, respectively. After mounting, a freshly isolated murine lung was ventilated and perfused with an open outlet for 15 min. A 20 ml reservoir containing the substance to be tested [EA (50, 100 nM)) or perfusion solution of decreased pH values (pH 6.0, 5.0 and 4.0)] replaced the perfusion solution and pH values were adjusted for 10 min in the external reservoirs. The outlet was closed and lungs were continuously perfused with or without FITC–dextran particles and monitored for an additional 50 min. Lung weight and tidal volume were constantly quantified during the experiment, while wet-to-dry weight ratios and invasion of FITC–dextran particles in the tissues were analyzed after the experiment.

### Validation of the IPVML for detection of acute lung toxicity induced by short drops of the pH

**Fig. 2:**
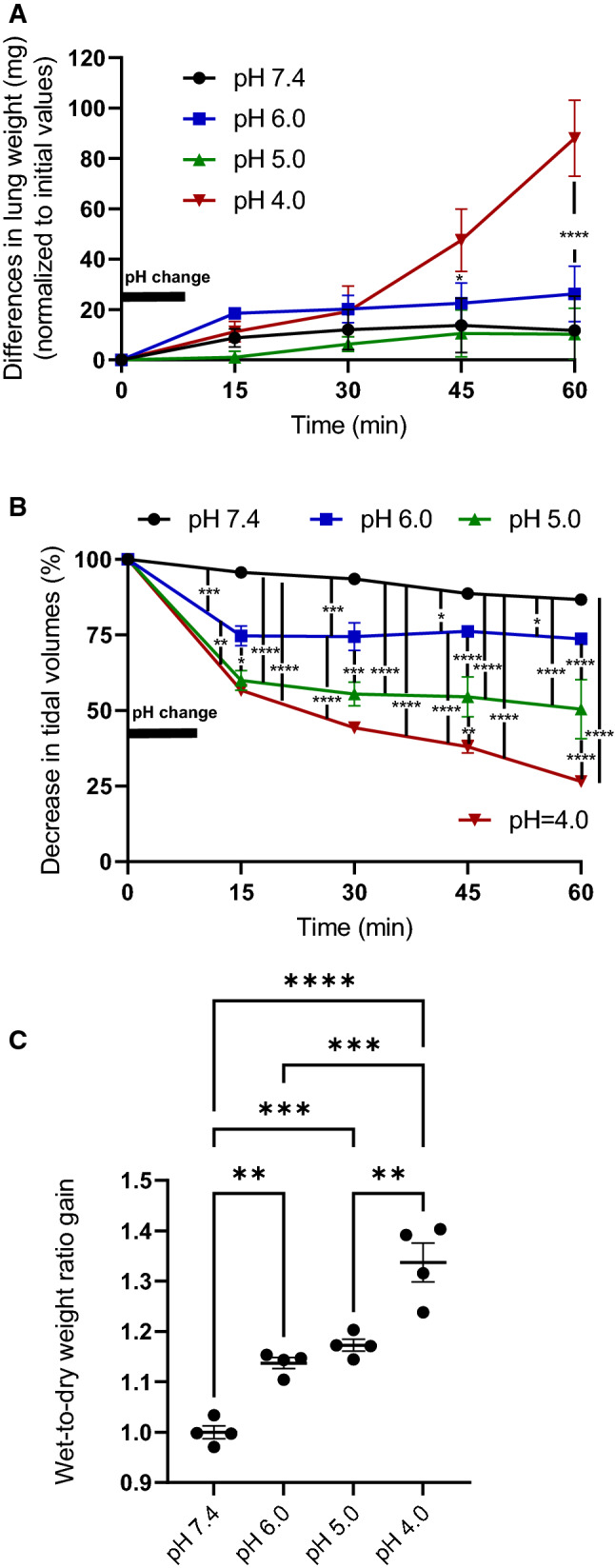
Lung weight gain after a short (10 min) drop of the pH to 6.0, 5.0 and 4.0 (**A**). Changes in tidal volumes after a short (10 min) drop of the pH to 6.0, 5.0 and 4.0 (**B**). Increases in wet-to-dry weight ratios after a short (10 min) drop of the pH to 6.0, 5.0 and 4.0 were quantified after the experiment (**C**). Data are means ± SEM. *p* values were calculated by two way ANOVA and are indicated by asterisks (*, *p* < 0.05; 2*, *p* < 0.01; 3*, *p* < 0.001, 4*, *p* < 0.0001)

To evaluate the sensitivity of the IPVML model, we applied solutions with a decreased pH (pH 6.0, pH 5.0 and pH 4.0). Significant increases in lung weight due to an acute formation of lung edema during the experiment were only detected for a short-time drop to pH 4.0, but not for pH 5.0 or pH 6.0 (Fig. 2A). A linear decrease in tidal volumes with dropping pH values was, however, obvious during the experiment (Fig. 2B). Wet-to-dry weight ratios after the experiment were linearly increased for transient drops to pH 6.0, pH 5.0 and pH 4.0 (Fig. 2C). Therefore, short-time changes in pH correlate with a linear increase in wet-to-dry weight ratios and a linear decrease in tidal volumes in the IVPML model. The experimental setup is, therefore, suitable and sensitive enough to detect acute lung edema in a linear fashion.

### Biological activity of (−)-englerin A (EA) before and after perfusion in the IPVML

**Fig. 3:**
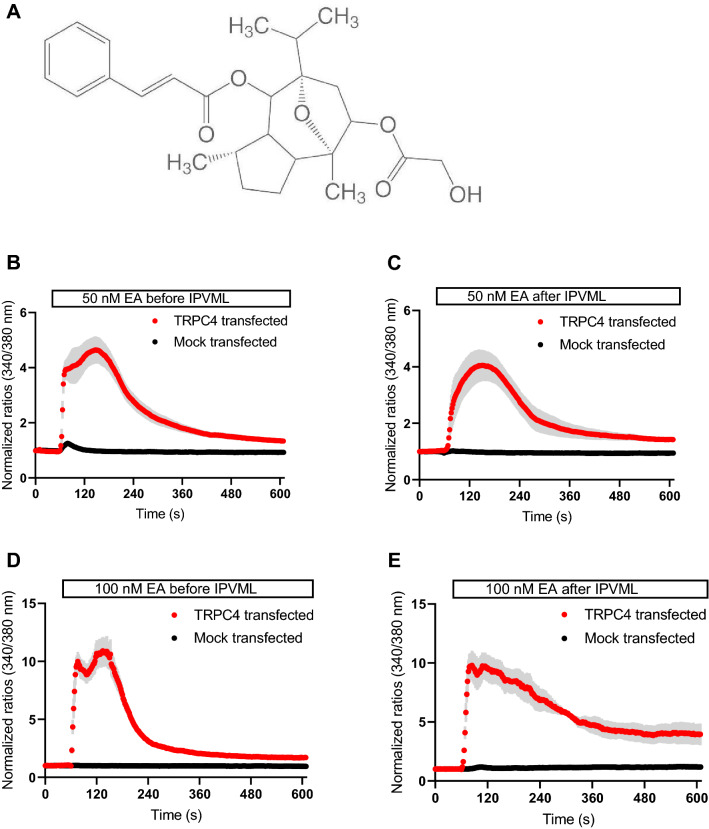
Structural formula of (−)-englerin A (EA, provided by the manufacturer, see www.carlroth.com, **A**) and Ca^2+^ imaging experiments of HEK 293T cells heterologously expressing TRPC4 channels (TRPC4 transfected) or mock-transfected controls after application of EA (50 nM, 100 nM) before and after 1 h perfusion in the IPVLM model. One representative experiment (n > 3 cells) out of three is shown (**B–E**)

The biological activity of EA (Fig. 3A) was quantified by Ca^2+^ imaging of HEK 293T cells heterologously expressing TRPC4 channels after transfection with a pIRES plasmid carrying cDNAs for TRPC4 and an enhanced green fluorescent protein (eGFP). EA (50 and 100 nM) before and after the experiment (1 h perfusion) was able to increase intracellular Ca^2+^ ([Ca^2+^]_i_) levels by opening TRPC4 channels and Ca^2+^-influx through the channel pore, while non-transfected cells show only minor changes in [Ca^2+^]_i_ (Fig. 3B–E). Our results indicate a preserved biological activity of EA after 1 h perfusion in the IPVML model.

### Evaluation of acute lung toxicity induced by EA in the IPVML

**Fig. 4:**
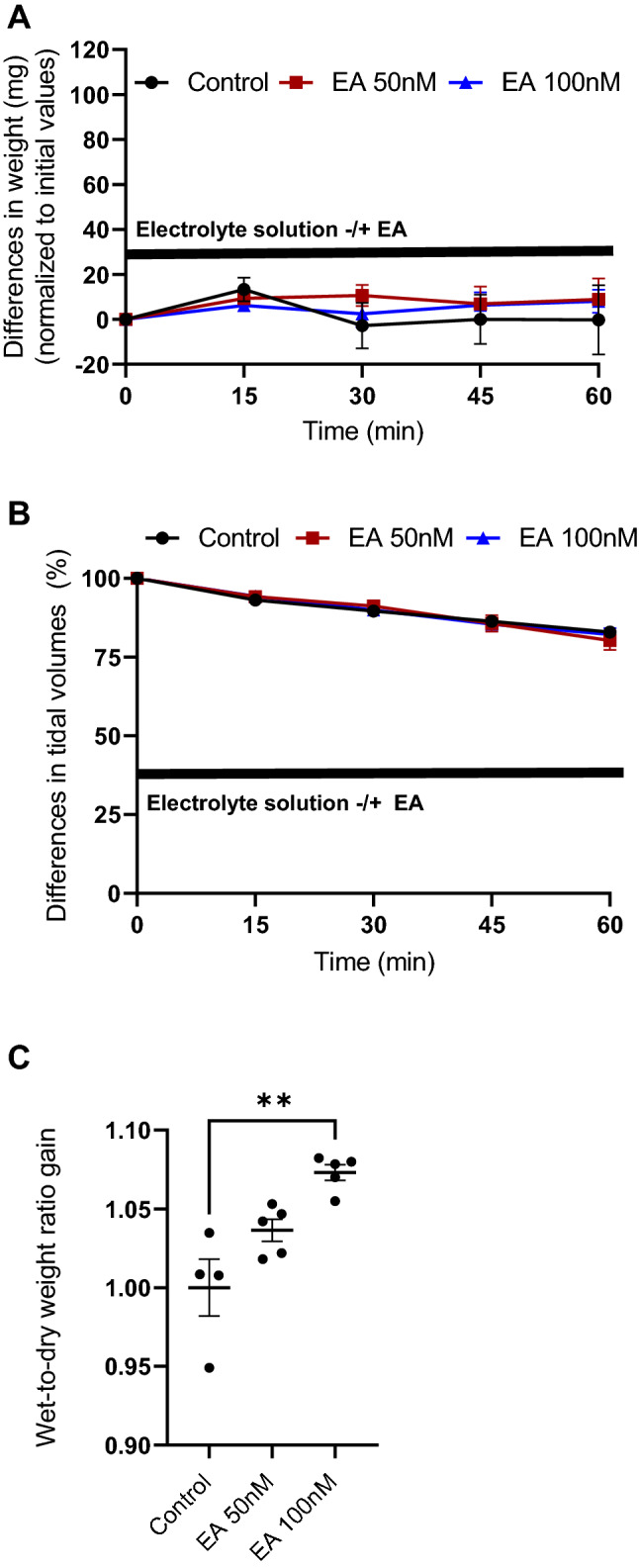
Lung weight gain after application of (−)-englerin A (EA, 50 and 100 nM) (**A**). Changes in tidal volumes after application of EA (50 and 100 nM) (**B**). Increases in wet-to-dry weight ratios after after application of EA (50 and 100 nM) (**C**). Data are means ± SEM. *p* values were calculated by two way ANOVA and are indicated by asterisks (*, *p* < 0.05; 2*, *p* < 0.01)

Changes in lung weight during 1 h perfusion of EA (50 and 100 nM) in comparison to electrolyte solution are depicted in Fig. 4A. No significant differences were observed. The same was true for the analysis of tidal volumes during the experiment (Fig. 4B). Wet-to-dry weight ratios showed, in comparison to short drops in pH, a small but significant increase after application of EA (100 nM), which was not detectable for the lower concentration (50 nM) (Fig. 4C).

### Quantification of lung permeability by tissue permeation of FITC–dextran particles in the IPVML

**Fig. 5:**
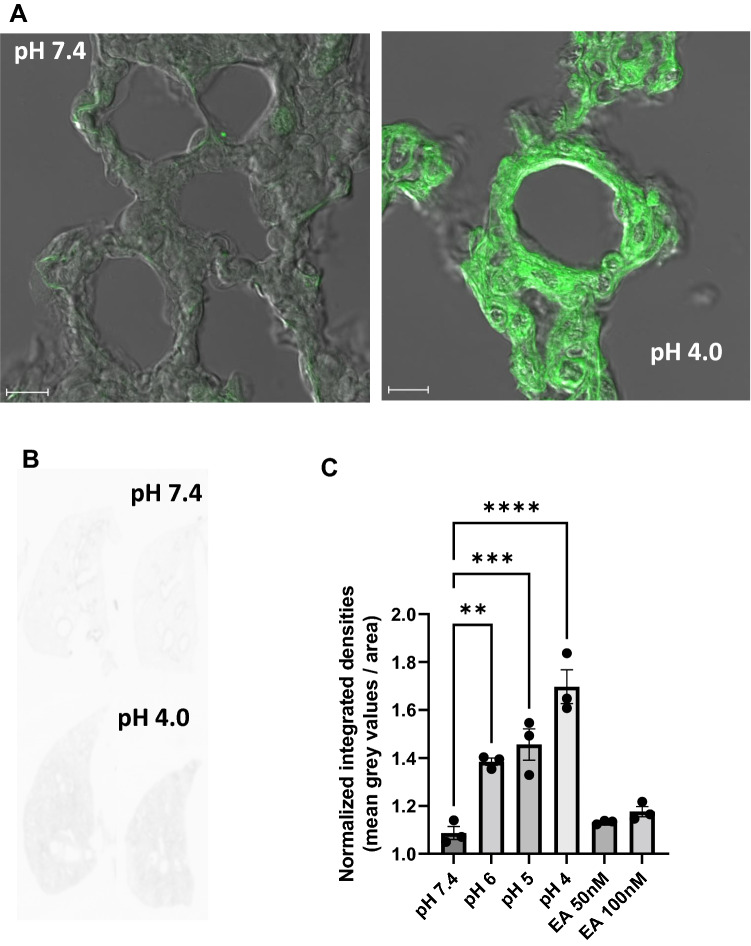
Tissue permeation of fluorescein isothiocyanate (FITC) dextrane particle after perfusion of lungs at pH 7.4 and after a short (10 min) drop of the pH to 4.0. Two representative overlays of FITC fluorescence and coresponding differential interference contrast (DIC) images showing lung tissues after perfusion of electrolyte solution (pH 7.4) and after a short (10 min) drop in pH (pH 4.0) are depicted (**A**). Two representative scans from total lungs at pH 7.4 and after a short (10 min) drop of the pH to 4.0 (**B**). Normalized integrated densities of lungs after a short (10 min) drop of the pH to 6.0, 5.0 and 4.0 or perfused with EA (50 and 100 nM) were normalized to lungs perfused with electrolyte solution at pH 7.4 and plotted as columns. Data are means ± SEM. *p* values were calculated by two way ANOVA and are indicated by asterisks (*, *p* < 0.05)

Endothelial and tissue permeabilty is significantly increased after exposure of toxicants from the vascular side. To quantify tissue permeation of liquids and invading cells fluorescein isothiocyanate (FITC)-coupled dextran particles are used in cell biology and in vivo animal experiments (Thorball 1981). To test if our model is also able to detect increases in tissue permeabilty in a linear fashion, we added FITC–dextran particles in the perfusion solution during the preflow and analysed whole lungs by confocal laser scanning microscopy and by a laser scanning platform. Tissues after a short drop to pH 4.0 incorporated a significant higher amount of FITC–dextran particle than tissues perfused with control solution of a pH 7.4 as shown in overlay images in Fig. 5A. To quantify fluorescence in tissues without a selection bias, we scanned whole lungs on a laser scanning platform as shown in Fig. 5B. Integrated fluorescent values normalized to values of non-perfused lungs are shown in Fig. 5C. Short-time pH drops caused a significant linear increase in incorporation of FITC–dextran particels compared to EA and electrolyte solution-only perfused mouse lungs. Therefore, this ex vivo lung model is also suitable to detect higher tissue permeability after application of toxicants by permeation of FITC–dextran particles.

## Discussion

EA is a promising anti-cancer compound inhibiting tumor growth of several kidney (Ratnayake et al. 2009) (Akbulut et al. 2015), breast cancer (Grant et al. 2019) and synovial carcinoma cell lines (Muraki et al. 2017). Its toxicological evaluation in rodents, however, is complicated for two reasons. Intravenous injections of EA (2 mg/kg body weight) were lethal to rats and after subcutaneous application EA was rapidly degraded into the inactive englerin B in mice and rats, while it remains stable in humans (Carson et al. 2015). Therefore, we choose the IPVML model as an ex vivo approach to evaluate acute pulmonary toxicity of EA independently from the mode of application and degradation. We first tested its sensitivity by short drops of the pH of the perfusion medium from pH 7.4 to pH 4.0. These pH changes can be observed in asthma patients after accumulation of CO_2_ in their blood due to their inability to exhale or by infusion of acids (Richardson et al. 1961). Moreover, metabolic acidosis and septic shock also decrease pH values in the blood (Mizock and Falk 1992). Significant linear increases were observed for wet-to-dry weight ratios (Fig. 2C), while tidal volumes decreased (Fig. 2B) after a stepwise drop in pH to 4.0. Lung edema formation quantified by an increase in lung weight during perfusion and ventilation were only detected after a drop to pH 4.0, while decreases to pH 5.0 and 6.0 did not induce immediate changes in lung weight (Fig. 2A). While small drops in pH to 6.5 did not change intracellular pH values significantly in endothelial cells of the rat aorta but increase intracellular NO concentrations (Capellini et al. 2013), large decreases in pH values may damage endothelial cells and increases endothelial permeability. As a consequence flooding of alevoli by protein rich fluid, increases lung weight and reduces tidal volumes. Therefore, the ex vivo IPVML model realistically maps the in vivo situation of acute lung toxicity by quantifying edema formation due to a transient decreased pH.

The biological activity of EA was tested before and after 1 h perfusion in the IPVML model by application to HEK 293T cells heterologously expressing TRPC4 channels or control cells. EA induced significant increases in [Ca^2+^]_i_ as already described (Akbulut et al. 2015) even after 1 h of perfusion in the IPVML model in comparison to control cells (Fig. 3A–D). As expected EA is still active and supposedly not degraded to the inactive englerin B in our perfusion solution as enzymes, such as esterases, which might degrade EA in rodents in vivo (Carson et al. 2015), may not be present in the IPVML model.

Tidal volumes and lung weights correlating with lung edema formation were not changed during the experiment (Fig. 4A, B) using EA for perfusion in concentrations of 50 and 100 nM, while wet-to-dry weight ratios after the experiments were significantly increased after application of 100 nM EA (Fig. 4C).

However, histological analysis of lung sections after perfusion with FITC–dextran particle revealed a similar linear increase in the incorporation of these particles in the lung tisues by transient drops of the pH, while both concentrations of EA were not able to change fluorescence intensities significantly (Fig. 5C). Therefore, a significant change in edema formation as detected in wet-to-dry weight ratios after perfusion with 100 nM EA was not confirmed in these experiments.

An overall comparison of the parameters collected in the IPVML model places tidal volumes, wet-to-dry weight ratios and FITC–dextran tissue distribution as the most sensitive values for detection of acute lung toxicity after a short pH drop, while lung weight changes during perfusion were only able to detect a major change in pH from 7.4 to 4.0.

In light of an expression of TRPC4 channels in the lung endothelium and their identified ability to increase endothelial permeabilty after activation of protease-activated receptors (PAR) by thrombin (Tiruppathi et al. 2002), our results with the highly specific TRPC4 activator EA are somewhat surprising. Except for changes in the wet-to-dry weight ratios after perfusion with 100 nM EA, we detected no differences in edema formation after application of EA in comparison to electrolyte solution as control. This discrepancy may be due to a close clustering of PAR together with TRPC4 channels in calveolar, such as compartments, which are not easily accessible for EA from the perfusate. This hypothesis needs to be further studied in the future.

In summary, the IPVML model is suitable to evaluate acute pulmonary toxicity of drug candidates by quantification of lung edema formation independently of drug application and metabolism. In the future numerous already established gene-deficient mouse models may also be used to identify toxicant sensors in the lung by this method. Moreover, this ex vivo model eliminates stress in mice after in vivo application of drugs and is, therefore, an important refinement according to the 3R ethical guidelines of animal experimentation.

## Abbreviations

[Ca^2^^+^]_i_: Intracellular Ca^2^^+^ concentration
EA: (−)-Englerin A
FITC: Fluorescein isothiocyanate
h: Hour(s)
IPVML: Isolated perfused and ventilated murine lung
min: Minute(s)
TRPC4/5: Classical transient receptor potential 4/5
